# Fully convolutional architecture vs sliding-window CNN for corneal endothelium cell segmentation

**DOI:** 10.1186/s42490-019-0003-2

**Published:** 2019-01-30

**Authors:** Juan P. Vigueras-Guillén, Busra Sari, Stanley F. Goes, Hans G. Lemij, Jeroen van Rooij, Koenraad A. Vermeer, Lucas J. van Vliet

**Affiliations:** 1 Delft University of Technology, Dept. of Imaging Physics, Lorentzweg 1, Delft, 2628CJ The Netherlands; 2 Rotterdam Ophthalmic Institute, Schiedamse Vest 160, Rotterdam, 3011BH The Netherlands; 3 The Rotterdam Eye Hospital, Schiedamse Vest 180, Rotterdam, 3011BH The Netherlands

**Keywords:** Convolutional neural networks, U-net, Sliding-window CNN, Fourier analysis, Specular microscopy

## Abstract

**Background:**

Corneal endothelium (CE) images provide valuable clinical information regarding the health state of the cornea. Computation of the clinical morphometric parameters requires the segmentation of endothelial cell images. Current techniques to image the endothelium in vivo deliver low quality images, which makes automatic segmentation a complicated task. Here, we present two convolutional neural networks (CNN) to segment CE images: a global fully convolutional approach based on U-net, and a local sliding-window network (SW-net). We propose to use probabilistic labels instead of binary, we evaluate a preprocessing method to enhance the contrast of images, and we introduce a postprocessing method based on Fourier analysis and watershed to convert the CNN output images into the final cell segmentation. Both methods are applied to 50 images acquired with an SP-1P Topcon specular microscope. Estimates are compared against a manual delineation made by a trained observer.

**Results:**

U-net (AUC=0.9938) yields slightly sharper, clearer images than SW-net (AUC=0.9921). After postprocessing, U-net obtains a DICE=0.981 and a MHD=0.22 (modified Hausdorff distance), whereas SW-net yields a DICE=0.978 and a MHD=0.30. U-net generates a wrong cell segmentation in only 0.48% of the cells, versus 0.92% for the SW-net. U-net achieves statistically significant better precision and accuracy than both, Topcon and SW-net, for the estimates of three clinical parameters: cell density (ECD), polymegethism (CV), and pleomorphism (HEX). The mean relative error in U-net for the parameters is 0.4% in ECD, 2.8% in CV, and 1.3% in HEX. The computation time to segment an image and estimate the parameters is barely a few seconds.

**Conclusions:**

Both methods presented here provide a statistically significant improvement over the state of the art. U-net has reached the smallest error rate. We suggest a segmentation refinement based on our previous work to further improve the performance.

## Background

Convolutional Neural Networks (CNNs) have considerably advanced the state of the art in computer vision in the last years. Although they were introduced 30 years ago [1], it was not until recently that improvements in computer hardware allowed large-scale training of more complex, deep networks [2]. Whilst the typical use of CNNs was aimed at learning classification tasks, segmentation is also a desired outcome in medical imaging. In 2012, Cireşan et al. [3] employed a typical classification architecture to perform tissue segmentation. They segmented neural membranes images from electron microscopy by using a CNN in a sliding-window setup such that in order to predict the class label of a target pixel, a local region (patch) around that pixel was provided as input. Although this strategy yielded great results (it won the ISBI 2012 challenge), it was computationally expensive and did not exploit the redundancy between overlapping patches. In 2015, Ronneberger et al. [4] proposed the U-net, which turned out to be a major contribution to the field of biomedical image segmentation. This network, an extension of a ‘fully convolutional network’ presented in a previous paper [5], had the benefits of faster training by introducing skip-layer connections between layers of the same resolution and by not using fully connected layers. U-nets accept the whole image as input and obtain good results with just a very few annotated images to train on, which made it win the ISBI 2015 challenge. In this paper we aim to adapt, improve, and evaluate a local sliding-window CNN (named SW-net) and a global fully convolutional U-net to segment corneal endothelium (CE) images obtained with specular microscopy.

The CE is a monolayer of closely packed and predominantly hexagonally-shaped cells on the posterior surface of the cornea. Endothelial cells are 4-6 *μ*m in height and 20 *μ*m in width [6], and they play a key role in maintaining an optimal state of corneal hydration [7], but they do not undergo mitosis in vivo. Instead, when cells are lost through age-related apoptosis or trauma, the remaining healthy cells grow and migrate to occupy the space of the lost cells. As a result, the CE cell architecture loses its hexagonal appearance. In young adults, the endothelial cell density is around 3000-3500 cells/mm^2^, but generally lower than 2000 cells/mm^2^ in elderly people [8]. If the cell density reaches a critical point due to trauma or eye diseases (around 500-700 cells/mm^2^), corneal edema occurs. Since edema leads to poor vision, corneal transplantation is usually the treatment in those situations.

Currently, three parameters are used to evaluate the health status of the endothelium: endothelial cell density (ECD), polymegethism (or cell variation, CV), and pleomorphism (or hexagonality, HEX). To correctly estimate the clinical parameters, an accurate segmentation of the cells is necessary. The current clinical standard technique to image the endothelium in vivo is non-contact specular microscopy, which is fast and non-invasive. However, images might appear blurred since this technology requires corneas to have a smooth endothelium surface [9]. In addition, noise, illumination distortions, and optical artifacts are commonly present in specular images.

Manual delineation of the cells is a very labor-intensive task. Existing commercial software for cell segmentation, usually provided by the microscope manufacturers, has limited performance. Several studies using specular microscopy have shown the inaccuracy of the automated analyses [10–13]. For instance, Luft et al. [14] compared four different non-contact specular microscopes in combination with their built-in segmentation software – models: EM-3000, Tomey; CEM-530, Nidek; CellChek XL, Konan; and Perseus, Bon Optic – in healthy eyes and eyes with corneal grafts, and concluded that all models (except Konan) significantly underestimated ECD in the subgroup of healthy eyes, whereas ECD was significantly overestimated in the corneal graft group for all models.

Several algorithms for in vivo corneal endothelial cell segmentation have been proposed in the last three decades. The early approaches (90s and early 00s) used simple methods, such as a combination of thresholding, skeletonization, Gaussian filtering, and morphological operations [6, 15, 16], shape dependent filters [17], and the seeded watershed algorithm [18–20] (each one using different morphological operations to place the seeds). These methods only provided relatively good results for high quality images and their clinical application was never evaluated. Moreover, many of them suggested the necessity of user interaction to correct errors. In contrast, new clinically applicable methods have been proposed in recent years: Foracchia and Ruggeri [21] developed an algorithm based on Bayesian shape models, which later evolved into a genetic algorithm by Scarpa and Ruggeri [22]; Sharif et al. [23] developed a hybrid model based on a combination of an active contour model (snakes) and a particle swarm optimization approach; Habrat et al. [24] proposed an algorithm based on directional filters, which was clinically evaluated along with other methods [25]; Al-Fahdawi et al. [26] suggested a method based on the watershed algorithm and Voronoi tessellations; Selig et al. [27] employed Fourier analysis and the seeded watershed algorithm in a stochastic manner to segment confocal images; and Vigueras-Guillén et al. [28] proposed a classifier-driven method to generate an accurate segmentation from an oversegmented image, using Selig et al.’s approach [27] to generate the oversegmentation. Among these methods, the ones including a comparison with their respective microscope’s estimates were significantly more accurate, yet some mistakes were still present.

Regarding the use of neural networks or CNNs to segment CE images, four algorithms were published in the last year. Fabijańska [29] proposed a feed-forward neural network with one hidden layer to segment 30 *ex vivo* endothelial images from phase-contrast microscopy (dataset published in [30]), achieving an error in cell number detection of 5% and a DICE [31] value of 0.85. Nurzynska [32] further improved the results on the same dataset by employing a CNN in a sliding-window setup, using a similar network as Cireşan et al. [3], and obtaining a precision of 93% and a DICE of 0.94. Phase-contrast microscopy yields *ex vivo* CE images of high quality, which cannot be compared with in vivo specular microscopy. In fact, we already solved that dataset, achieving a segmentation error in only 0.28% of the cells and an average error in the clinical parameter estimates of less than 0.4% [28]. Katafuchi et al. [33] also used a CNN in a sliding-window setup to segment human endothelium in vivo, although they did not specify the imaging technology. They also employed a similar network as Cireşan et al. [3], and they achieved an error rate of 12%. Since neither of these two papers did a clinical evaluation, no further comparison can be described here. Finally, Fabijańska [34] was the first to apply the U-net to specular images, although using patches as input instead of whole images. She achieved a DICE of 0.85, an AUC (area under the ROC curve) of 0.92, and the error in the clinical parameters were 5.2% in ECD, 11.93% in CV, and 6.2% in HEX. In other image modalities, different neural networks architectures have been used for image segmentation, such as the use of fuzzy deep neural networks for brain MRI images [35] in order to extract information from both fuzzy and neural representations. Whereas the use of these sophisticated architectures in CE images has not been studied yet, it does not seem to be necessary given the rather low complexity of the cell patterns in CE images.

In summary, two main approaches have been exploited when using CNNs to segment endothelial cell images: via pixel classification (sliding-window setup, SW-net), or via direct segmentation (U-net). Here, we aim to clarify which approach is more optimal, proposing and evaluating two end-to-end solutions to segment in vivo CE images acquired with specular microscopy. Specifically, we use a preprocessing technique, a contrast limited adaptive histogram equalization (CLAHE) [36], to enhance the contrast of the images, and evaluate whether any image normalization is beneficial; we propose a modification of the image labels to make them probabilistic instead of binary, which improves the performance; we evaluate several implementation choices of the CNNs; and we suggest a postprocessing method to the CNN output in order to create the final segmented images.

This paper is organized as follows. In the “Results” section we evaluate the two networks in three ways: the performance of the CNNs and the importance of certain implementation details; the segmentation after applying the postprocessing method, reporting the distance to and similarity with the gold standard, as well as the percentage of correctly detected cells; and the accuracy of the estimated clinical parameters. In the “Discussion” section, we highlight the main findings and compare the results with some of the aforementioned methods. In the “Conclusions” section, we summarize the relevance of this study. Finally, in the “Methods” section, we describe the dataset, we illustrate the two networks, highlighting the changes we introduce, and we describe the pre- and post-processing techniques in detail, as well as all the metrics and statistical analysis employed.

## Results

### Evaluation on the CNN performance

#### Preprocessing method

Our experiments showed two main conclusions: (1) networks fed with raw images took slightly more time to converge, especially for SW-net; (2) either enhancing or standardizing/normalizing the images did not lead to prominent improvements in the performance (Table 1).

**Table 1:** Accuracy and AUC from the test fold for different types of preprocessing methods in both networks

Method	Accuracy	AUC
SW-net		
Raw	95.45	0.9932
Normalize	95.49	0.9933
Standardize	95.54	0.9937
CLAHE	95.82	0.9938
CLAHE+Standardize	95.88	0.9935
U-net		
Raw	97.65	0.9958
Normalize	97.67	0.9954
Standardize	97.64	0.9956
CLAHE	97.63	0.9957
CLAHE+Standardize	97.65	0.9953

SW-net provided higher accuracy when using CLAHE but similar AUC, which suggested that enhancing the images helps in the classification of those pixels whose *p* is closer to 0.5, but no significant changes occur in the proper edge (*p*=1) and body (*p*=0) pixels. For U-net, the differences were even smaller. In fact, the case with raw images provided the largest AUC. This suggested that U-net does not need any type of preprocessing to perform at its best.

In conclusion, we selected the type of preprocessing with the largest AUC: raw images for U-net, and CLAHE for SW-net.

#### Over-fitting, elastic deformations, and dropout layers

We observed that over-fitting was an important problem in training U-net. We could either tackle the issue by adding dropout layers (our approach), by using more data augmentation (elastic deformations), or both.

**Fig. 1:**
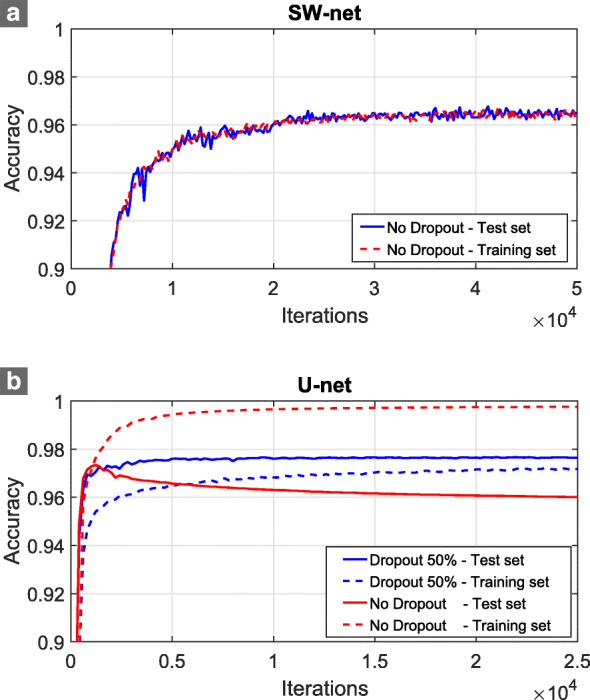
Effect of using dropout layers for SW-net (**a**) and U-net (**b**), where accuracy is plotted as a function of iterations. Note that SW-net required more iterations in order to stabilize its accuracy

While elastic deformations could create an artificially large training set, dropout layers were already optimal, removing any effect of over-fitting in U-net and increasing the accuracy (Fig. 1b). If elastic deformations were added on top of that, the accuracy decreased from 97.65 to 97.22, which made us discard that approach.

In contrast, SW-net was not affected by over-fitting (Fig. 1a). In fact, the network diverged and classified all pixels as cell body when dropout layers with a drop rate of 50% were added. Furthermore, we investigated whether substituting the global averaging layer for a fully connected layer had any effect in performance. We observed that over-fitting was also not present when using a fully connected layer of 200 neurons (as Cireşan et al.’s network [3]), but performance degraded (Table 2).

**Table 2:** Receptive field (RF, in pixels), accuracy, and AUC from the test fold for different types of filter sizes, number of filters, depth of the network (resolution steps), using class weighting or binary labels (for U-net), and patch size (for SW-net)

Method	RF	Acc.	AUC
SW-net			
** Patch 64pix, 32 filters of 3x3**	**61**	**95.82**	**0.9938**
Default, Fully Connected Layer	61	94.97	0.9916
Patch 96pix, 32 filters of 3 ×3	61	94.03	0.9888
Patch 96pix, 32 filters of 4 ×4	91	95.39	0.9931
U-net			
32 filters of 3 ×3, 4 steps	61	97.55	0.9949
32 filters of 3 ×3, 5 steps	125	97.62	0.9955
**32 filters of 4x4, 4 steps**	**91**	**97.65**	**0.9958**
32 filters of 5 ×5, 4 steps	121	97.46	0.9954
32 filters of 4 ×4, 3 steps	43	97.48	0.9951
32 filters of 4 ×4, 5 steps	187	96.92	0.9939
16 filters of 4 ×4, 4 steps	91	97.32	0.9951
64 filters of 4 ×4, 4 steps	91	97.61	0.9956
Default, weighted class	91	96.65	0.9958
Default, binary labels	91	93.92	0.99 19

Best performing (default) networks are indicated in bold

Figure 1 also shows the difference between both networks in terms of stability and convergence. Training U-nets yields much faster convergence and is more stable than training SW-nets. The latter shows a relatively large accuracy fluctuation, probably due to the large variation between patches. However, it is worth noting that, for the SW-net, we only sampled randomly 200 batches (25600 patches) from the test set every 200 training iterations, whereas the whole test set (10 images) was evaluated for the U-net at the same iterations. Using the whole test set for SW-net would entail to evaluate 12 million patches, which was extremely expensive computationally if evaluated so frequently. This was only done once the training was finished. Regarding the results for the training set in Fig. 1, they indicate the average accuracy in the 200 training batches previous to each test evaluation. Since batches in both networks had similar amount of data, it is possible to conclude that U-net is more stable. Nonetheless, both networks did not show any type of performance degradation as the number of iterations increases.

#### Receptive field and filter size

A key discrepancy between the two networks was the difference in receptive field size (Table 2). It is believed that a cell only has a direct effect in the shape of its adjacent cells. Indeed, it was observed a long time ago how the endothelial cells elongate and pull their neighboring cells when they need to cover a large space of dying cells [37]. Hence, it was expected that, in order to classify one pixel, only the shape and intensity information of the neighboring cells was required. Given that the average cell diameter is 25-30 pixels, a receptive field of 75-90 pixels would be optimal. Indeed, our experiments suggested that for U-net: the performance degraded when decreasing the receptive field, either by using filters of 3 ×3 or removing one resolution step, but also when increasing the receptive field, either by using larger filters of 5 ×5 or adding another resolution step (Table 2). Based on the cell size, more than 5 resolutions steps would be counterproductive, as cells would be unrecognizable at the last resolution (2^5^=32>average cell size).

**Fig. 2:**
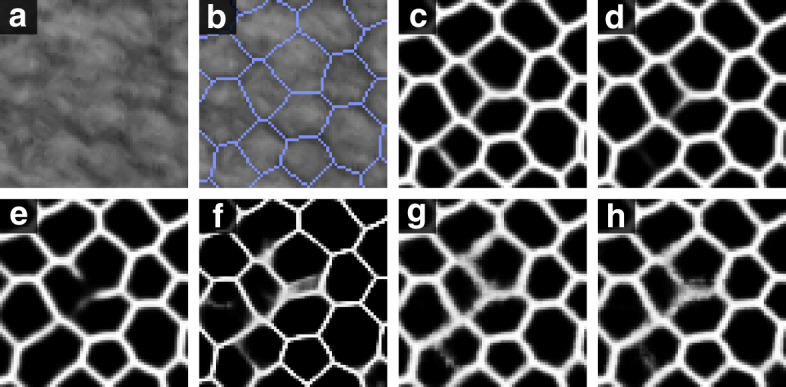
**a** Small, blurred area of a specular image (size 68 ×68 pixels) where the identification of small cells is difficult. **b** The gold standard (in blue) superimposed on the intensity image. **c** U-net output for a filter size of 4 ×4. **d** U-net output for a filter size of 3 ×3 with similar receptive field. **e** U-net output for a filter size of 5 ×5 with similar receptive field. **f** Default U-net output for a filter size of 4 ×4, but using the original binary labels. **g** SW-net output for a filter size of 4 ×4. **h** SW-net output for a filter size of 3 ×3

It could be argued that a different network composition with different filter sizes, but reaching the desired receptive field, would also be optimal. To evaluate this, we built networks reaching comparable receptive fields: for the 3 ×3 filters, we added another convolutional layer at each resolution step of the contraction path (receptive field of 93 pixels); for the 5 ×5 filters, we removed the last convolutional layer of the contraction path (receptive field of 89 pixels). Still, accuracy and AUC for the network using filters of 4 ×4 were always slightly higher (data not included). Moreover, visual evaluation indicated that filters of 4 ×4 (Fig. 2c) were somehow better than 3 ×3 (Fig. 2d) or 5 ×5 (Fig. 2e) in segmenting complex areas where the contrast was low. We believe this is due to the transposed convolutional layers and their problems in handling filter sizes not divisible by the stride, as discussed in the “Methods” section. This hypothesis was reinforced when the same experiment was done using SW-net, where no transposed convolutions were present, obtaining similar noisy results for both filter sizes, 3 ×3 and 4 ×4 (Fig. 2g and h).

In comparison with U-net, SW-net generated a ‘grainy’ effect in those complex areas. Moreover, it was observed that increasing the patch size to 96 pixels did not improve the performance (Table 2). Thus, the receptive field of SW-net was significantly smaller than that of U-net. This might be linked to the inherent nature of the patch-based approach, where increasing the patch size also increases the variation between patches, which in turn would take higher efforts for the CNN to distinguish patches of different classes.

Finally, we also tested the number of filters in U-net, halving or doubling them, obtaining slightly less accuracy in both cases (Table 2). In general, we observed that modifying the depth and width of our U-net did not drastically degraded the performance. Considering that the postprocessing corrects some mistakes and enhances the final segmentation, most probably all these networks would give similar clinical estimates.

#### Weighted classes and binary labels

Two distinctive decisions were taken when designing the network: not weighting the classes for U-net, and using probabilistic labels instead of the binary gold standard images.

Weighting the classes did not change the AUC in U-net, but the accuracy decreased (Table 2). The visible effect was slightly thicker edges, which in turn provided higher sensitivity* (0.9954 instead of 0.9940), but lower precision* (0.9907 instead of 0.9938).

On the contrary, the use of binary, weighted labels was clearly a mistake in terms of performance (Table 2). Furthermore, it created a ‘halo’ effect in complex areas (Fig. 2f), with no clear intensity pattern, which would create many artifacts in the postprocessing step.

#### The effect of the amount of training data

**Fig. 3:**
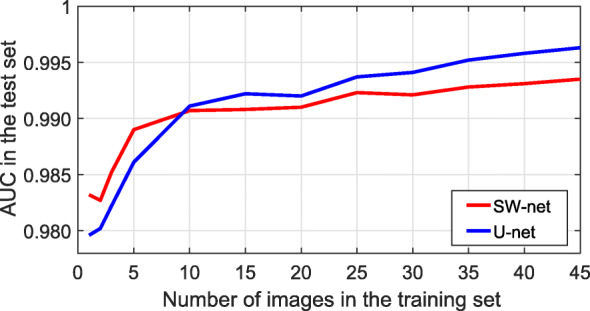
Network performance (AUC) based on the number of training examples

Large training sets are important to achieve good results in CNNs. To evaluate this, we defined an experiment where the training set was comprised of the following number of images, *n*_*training*_= [ 1,2,3,5,10,15,20,25,30,35,40,45], while the remaining images were assigned to the test set. AUC was retrieved for each case (Fig. 3). The experiment showed the following: (1) although over-fitting was present when less than 25 training images were used, no degradation in the performance of the test set was observed; (2) both networks could perform reasonably well with just one training image; (3) the performance of U-net improved more acutely than that of SW-net as more training images were included. In summary, this experiment suggested than building a larger training dataset might be the best choice to improve the overall performance.

#### Comparison between U-net and SW-net

**Fig. 4:**
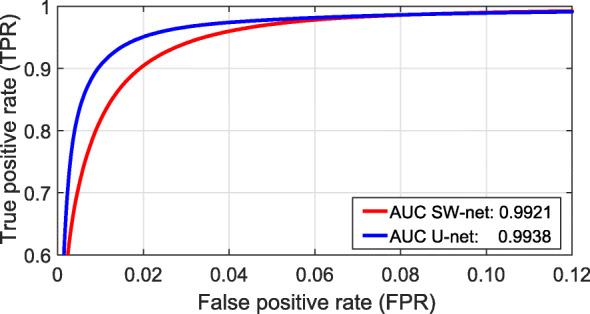
The ROC curves (zoomed) and the corresponding AUC values for both networks

Finally, we tested all images in both networks by employing a 5-fold cross-validation, using their respective best design parameters indicated above. The computed metrics clearly showed a higher performance for U-net (Table 3), with a considerably larger accuracy and precision. The ROC (Receiver Operating Characteristic) curves are displayed in Fig. 4.

**Table 3:** Accuracy, AUC, precision*, sensitivity*, and specificity* from all images (i.e. using a 5-fold cross-validation) in both networks, SW-net and U-net

	Acc	AUC	PRE*	SEN*	SPE*
SW-net	95.48	0.9921	0.9585	0.9906	0.9914
U-net	97.33	0.9938	0.9855	0.9892	0.9971

### Evaluation after applying postprocessing

The postprocessing method was especially effective when the cell size in the image was rather regular, as it detected weak edges in the CNN output and ‘filled’ discontinuities in the visual appearance of some edges (Fig. 4, green arrows). On the contrary, it sometimes reinforced weak, false edges in large cells (Fig. 6k, red arrow) or smoothed away small cells in images with a large variation in cell size (Fig. 6o and q, blue arrows). Furthermore, it was exceptionally beneficial for SW-net, as it corrected the ‘grainy’ edges. In Fig. 6, we reported the CNN output and final segmentation for three representative examples, along with the segmentation of the microscope’s built-in software. The gold standard images were not included, but instead the errors were indicated with red or blue arrows.

The modified Hausdorff distance (MHD) [38] indicated very low values for both networks (Table 4), which is in favor of concluding we achieved a very precise segmentation. To compare both networks, we applied the Wilcoxon signed-rank test since neither of both passed the Shapiro-Wild normality test (*p*<0.0001), achieving a statistically significant difference in favor of U-net (*p*<0.0001).

**Table 4:** Average MHD (±SD), average DICE (±SD), and percentage of over- (OC) and under-segmented (UC) cells, in both networks (SW-net and U-net), for *α*=1

Network	MHD	DICE	OC (%)	UC (%)
SW-net	0.30 ±0.09	0.978 ±0.006	0.537	0.382
U-net	0.22 ±0.04	0.981 ±0.003	0.220	0.260

The DICE metric [31] showed higher values for U-net (Table 4). Wilcoxon signed-rank test was also applied since the SW-net distribution did not pass the Shapiro-Wild normality test (*p*<0.0001), achieving a statistically significant better performance for U-net (*p*<0.0001).

Regarding the number of over- and under-segmented cells, U-net correctly segmented 99.52% of the cells. In contrast, SW-net achieved 99.08% success rate (Table 4). The distributions of ‘percentage of correctly segmented cells’ from both assessments failed the Shapiro–Wilk normality test (*p*<0.0001). The Wilcoxon signed-rank test indicated a statistically significant difference in favor of U-net (*p*=0.0006).

**Fig. 5:**
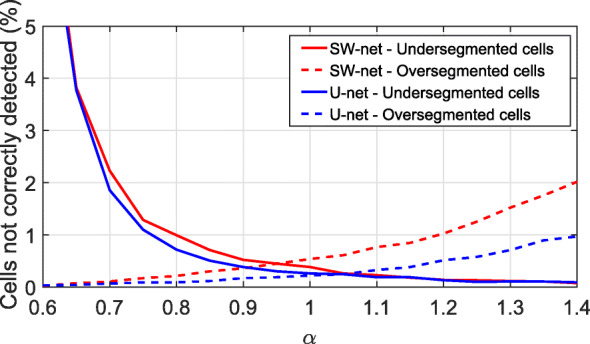
Percentage of wrongly detected cells (both, under- and over-segmented cells) for different scaling values (*α*) applied to *f*^∗^, for both networks

**Fig. 6:**
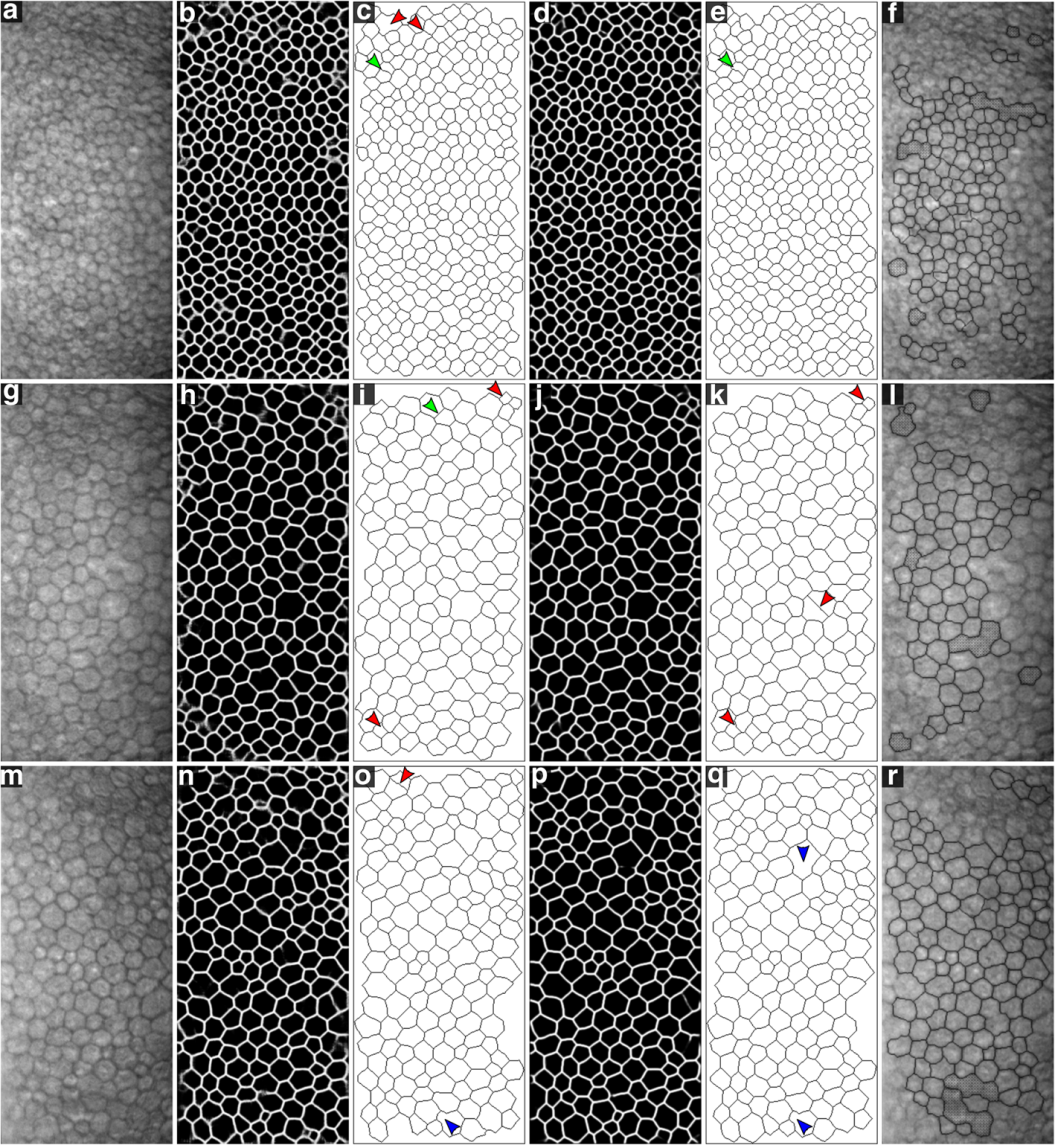
Three representative examples (high ECD in **a**-**f**, low ECD in **g**-**l**, high CV in **m**-**r**) for both networks. (**a**,**g**,**m**) Intensity images. (**b**,**h**,**n**) Outcome of the SW-net. (**c**,**i**,**o**) Segmentation after postprocessing of the SW-net outcome. (**d**,**j**,**p**) Outcome of the U-net. (**e**,**k**,**q**) Segmentation after postprocessing of the U-net outcome. Green arrows indicate true edges that were weak in the CNN output but detected by the postprocessing. Blue arrows denote true edges that were missed by the postprocessing, either because they were weak edges or because a small cell surrounded by large cells was smoothed away. Red arrows indicate false edges and mistakes in general. (**f**,**l**,**r**) Segmentation provided by the Topcon microscope’s built-in software

Furthermore, we evaluated the robustness of the postprocessing method by adding a scaling factor (*α*) to the estimated characteristic frequency, *σ*=*k*_*σ*_/(*αf*^∗^) (see “Methods” section). Specifically, we evaluated the method for both networks and values of *α* between 0.60 and 1.40 in steps of 0.05 (Fig. 5). Overall, both approaches yielded optimal results for values of *α*≈1, but the error for SW-net rose much faster as *α* increased. In comparison with the Topcon output segmentation (Fig. 6f, l and r), both our methods did significantly better, detecting all the cells in the image (roughly 70% more cells than Topcon).

### Evaluation on the clinical parameters

The clinical parameters for both methods were determined from the final segmentation results and compared to the corresponding values calculated based upon the gold standard. The same algorithm for parameter estimation was used in all sets, including Topcon’s segmentation images. For all images, only the cells covered by the area of the gold standard were included for the parameter estimation. The only exception was Topcon’s segmentation, since the microscope’s software did not provide any cell segmentation beyond the segmented area (Fig. 6). In that set, the gold standard covered twice its segmented area.

The clinical parameters were defined as follows. For cell density,

1 $$ \text{ECD} = \frac{{\sum\nolimits}_{i=1}^{n} S_{i}}{n},  $$

where *n* denotes the number of cells, and *S*_*i*_ the area (in pixels) of the *i*th cell, defined as *S*_*i*_=*B*_*i*_+*E*_1_/2, where *B* is the cell body and *E* the cell edge. Polymegethism was defined as

2 $$ \text{CV} = 100\%\frac{1}{\bar{S}}\sqrt{\frac{{\sum\nolimits}_{i=1}^{n} \left(S_{i}-\bar{S}\right)^{2}}{n}},  $$

where $\bar {S}$ stands for the average cell size. Finally, pleomorphism was defined as

3 $$ \text{HEX} = 100\%\frac{n_{hex}}{n},  $$

where *n*_*hex*_ denotes the number of six-sided cells.

**Fig. 7:**
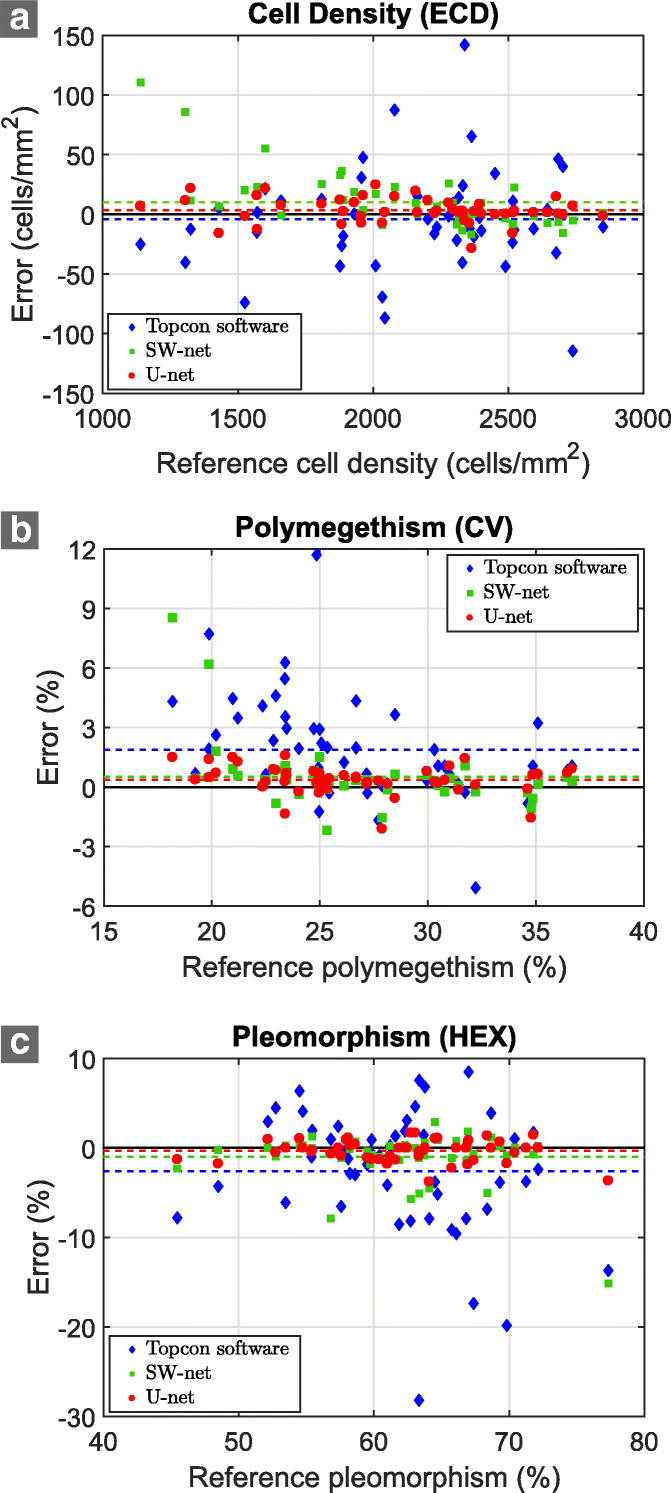
Estimates of the clinical parameters in both networks. The x-axis indicates the value for the gold standard, and the y-axis indicates the error computed as the difference between the network estimates and the gold standard estimates. Each point corresponds to one image in the dataset (U-net in red, SW-net in green, and Topcon in blue). The mean value of the error for each set is drawn with a discontinuous line. **a** Cell density (ECD). **b** Polymegethism (CV). **c** Pleomorphism (HEX)

The estimation error was defined as the difference between the estimated value and the gold standard value. The absolute error was defined as the absolute difference. Note that, for polymegethism (Fig. 7b) and pleomorphism (Fig. 7c), the parameter values were provided as a percentage, and the error was the difference of the percentages. The mean value and standard deviation (SD) of those estimation errors are indicated in Table 5. To statistically evaluate the precision, we used the SD of the error, whereas the mean absolute error was employed to evaluate the accuracy.

**Table 5:** Mean and standard deviation of the estimation error of the clinical parameters for both networks and Topcon microscope built-in software

Dataset	ECD (cells/mm^2^)	CV (%)	HEX (%)
Error			
Topcon	−4.1±41.7	1.9±2.6	−2.2±7.2
SW-net	9.9±23.1	0.5±1.6	−0.7±2.0
U-net	3.2±10.1	0.4±0.7	−0.2±1.0
Absolute error			
Topcon	29.8±29.6	2.3±2.2	5.3±5.3
SW-net	14.9±20.2	0.8±1.4	1.6±2.5
U-net	7.8±7.2	0.6±0.5	0.9±0.8
	ECD (%)	CV (%)	HEX (%)
Relative error			
Topcon	1.3±1.4	10.1±9.0	8.0±8.9
SW-net	0.8±1.3	3.6±7.3	2.1±2.9
U-net	0.4±0.4	2.8±2.6	1.3±1.0

Error and absolute error are computed as the difference (and absolute difference) between estimates and gold standard values. Relative error is computed as the percentage of the absolute error with respect to the gold standard values

The statistical analysis between U-net and Topcon indicated a significantly better precision and accuracy in all parameters for U-net (*p*<0.0001). For SW-net, the statistical analysis also indicated a significantly better precision (*p*<0.0001, *p*=0.0002, and *p*<0.0001 for ECD, CV and HEX, respectively) and a significantly better accuracy (*p*=0.0054, *p*<0.0001, and *p*<0.0001 for ECD, CV and HEX, respectively) than Topcon for all parameters.

Finally, we compared U-net against SW-net. The statistical analysis denoted a significantly better precision for U-net in all parameters (*p*<0.0001). The analysis also showed a significantly better accuracy in ECD for U-net (*p*=0.013) and HEX (*p*=0.048), but comparable for CV (*p*=0.30).

One of the main differences between SW-net and U-net was the robustness of U-net against images of different cell density. Indeed, SW-net tends to overestimate ECD as ECD decreases, whereas the ECD error for U-net is rather constant regardless of the cell density (Fig. 7a). This problem of SW-net might be explained by the large percentage of images of high ECD in the dataset, which in turn might lead the network to infer that cells are ‘normally’ of a small size. Interestingly, U-net can overcome this drawback, probably due to the fact that U-net can exploit the overlapping features between nearby pixels. Nonetheless, a more inhomogeneous and larger dataset would certainly improve this.

Clinically, it is more important to achieve better precision than accuracy, as the latter could be mitigated by adding a bias to all measures. Moreover, it is desired to obtain more precise, accurate estimates in the images with low ECD, as those are the cases where clinical decisions are more critical. In this sense, U-net is preferred over SW-net.

## Discussion

All the experiments regarding the CNNs architectures clearly indicated a quantitatively better performance in U-net. In contrast, the qualitative results were quite similar for the two networks, with only subtle differences, such as the ‘grainy’ effect on the SW-net output (Fig. 2). Overall, SW-net did not detect more false edges than U-net (Fig. 6), but the presence of blurred, faded edges in SW-net was manifest. Interestingly, those subtle differences had a significant effect in the biomarkers estimation. This highlights the importance of the postprocessing method, which in our case was designed to minimize those problems. A simpler postprocessing approach, such as thresholding and skeletonization, could potentially create many small false cells, sometimes of just a few pixels. This would require to define morphological operations ad hoc that would remove them. Given the large variation in cell size between images – or even in the same image (Fig. 6m) –, such operations would be prone to mistakes. In this respect, our postprocessing method does not require to define or tune any variable. Indeed, the 1D radial magnitude of the 2D Fourier Transform (FT) of the CNN output shows a clearly distinctive peak (Fig. 9b), which makes it easy to estimate the most common cell size in the image and adapt the Gaussian smoothing filter of the postprocessing to that size. The only drawback of this approach occurs when an image shows a large variation in cell size (as in Fig. 6m), where very small cells can be smoothed away (Fig. 6n-q). As we showed in Fig. 5, adding a scaling factor to create a thinner smoothing filter does not reduce the overall error in cell detection since oversegmented cells would rapidly increase if *α* is increased. However, we could tackle this problem by employing a refinement method. In our previous work [28], we performed the segmentation of CE images by employing a merging method that is applied to oversegmented CE images. There, we defined several features based on cell size, shape, and intensity, which were used to identify and remove false edges. Moreover, we showed how the errors mainly originated from wrong edge delineations in the oversegmented images and that the method was robust against a high degree of oversegmentation [39]. For those reasons, both methods could be combined in order to provide an even better performance. In this sense, the aforementioned problem could be simply solved by reducing the filter *σ* in order to generate a small degree of oversegmentation, and afterwards applying the merging method from our former study [28] (this refinement method was not tested in this paper).

Regardless of this suggestion for refinement, the currently proposed method achieves a relative average error in U-net of 0.4% in ECD, 2.8% in CV, and 1.3% in HEX. When comparing the relative error of CV and HEX in both networks with the Topcon estimates (Table 5), the improvement is outstanding, reducing the error in less than one third. In comparison with Fabijańska’s U-net paper [34], our U-net error is more than 4 times smaller. We believe that this large difference is not only due to the result of changes in the U-net architecture, but also due to the use of probabilistic labels in combination with a more sophisticated postprocessing method.

In comparison with other methods from the literature described in the “Background” section, we either achieved the smallest error rate in biomarker estimation and/or the smallest error in segmentation accuracy (only a few papers performed a full clinical evaluation). For instance, Scarpa and Ruggeri [22], who developed an algorithm that mimics biological evolution in order to detect the endothelial cells in specular microscopy images, achieved a relative average error of 0.6% in ECD, 5.33% in CV, and 3.11% in HEX; Selig et al. [27], who employed stochastic watershed to segment endothelial cells in confocal microscopy images, obtained a relative average error of 4.2% in ECD, 22.3% in CV, and 14.4% in HEX; or in our previous work regarding the merging method [28] we achieved an error of 0.8% in ECD, 4.5% in CV, and 3.9% in HEX. While the current work clearly indicates that we have achieved state-of-the-art results, the same dataset should be evaluated in all the previous proposed methods in order to validate that conclusion.

Finally, it is important to highlight that we evaluated a dataset of relatively healthy endothelial cell layers, whose main common factor – besides all being from glaucomatous eyes – was the old age of the subjects. Whereas these cases are the most commonly observed in the clinic, several cornea diseases, such as Fuchs’ dystrophy syndrome, bullous keratopathy, or keratoconus, provide heavily blurred, noisy specular images, sometimes with large portions of the image out of focus. Further work would be required to assess the performance of the proposed method in such cases. Moreover, it would be beneficial to develop a method that could automatically select the region of interest in the images from where to estimate the biomarkers, discarding the excessively blurred or unfocused areas. Currently, this is manually performed by the user.

## Conclusions

We have presented and evaluated two end-to-end methods for segmenting CE images, a global approach based on U-net and a local approach based on a sliding-window CNN (named SW-net). We have demonstrated excellent results with both approaches, outperforming the current segmentation that the microscope’s built-in software provides. Overall, U-net is the preferred approach, as it provides higher accuracy/precision and faster convergence in network training.

Up to now, the inability of providing an accurate segmentation made it difficult to use morphological biomarkers (CV or HEX) in clinical studies with large amount of data, even though it was observed decades ago that there is a direct link between these biomarkers and certain diseases [40, 41]. Indeed, cell density is currently the only endothelial biomarker used in the majority of clinical studies due to the limited accuracy of the current segmentation techniques. Deep learning now opens new opportunities to further analyze a large number of endothelial images.

## Methods

### Materials

The dataset contains 50 corneal endothelium images from the central cornea of 50 glaucomatous eyes, imaged with a non-contact specular microscope (SP-1P, Topcon Co, Japan). They are part of an ongoing study in The Rotterdam Eye Hospital regarding the implantation of a Baerveldt glaucoma drainage device.

Glaucoma is a condition related to the buildup of pressure inside the eye, which can eventually damage the optic nerve. In primary open-angle glaucoma (POAG), the eye cannot properly drain the aqueous humor through its drainage system, whereas in primary angle-closure glaucoma (PACG) the iris blocks the entrance of the drainage system. In PACG, surgical intervention is usually required to remove the blockage. In POAG, eye drops are the first treatment option in mild cases, either to reduce the formation of fluid in the eye or increase the outflow, but surgical intervention is usually considered when these treatment modalities have proven ineffective. Trabeculectomy is a common procedure, which consists of a small hole in the sclera, covered by a thin trap-door, which makes it possible to drain the aqueous humor out of the eye. However, scarring may lead to failure of the trabeculectomy. Therefore, but also because of other possible complications with trabeculectomies, glaucoma drainage devices are often preferred over trabeculectomy. Indeed, in refractory cases, the success rates five years postoperatively of Baerveldt implants are higher than those of trabeculectomies [42].

A common postoperative complication after implantation of a Baerveldt (or similar glaucoma drainage) device is a change in the CE, in both cell count and cell shape [43, 44], due to the proximity of the device’s tube. In the study currently ongoing in The Rotterdam Eye Hospital, eyes were imaged before and after the implantation of the device. Here, we focused on solving the cases prior to the implantation, which let us assume that the CE was only affected by the natural aging process. Indeed, it has not been observed that glaucoma has any direct effect in the morphology of the CE cells. In our dataset, the average age is 64.8±9.2 (mean ± SD). Our dataset showed a large variability in cell size and morphology, with a range of 1100-2800 cells/mm^2^ in ECD, and 18-36% in CV, and 44-74% in HEX.

Each image covers an area of 0.25 mm × 0.55 mm and was saved as 8-bits grayscale images of 240 ×528 pixels. According to the manufacturer, pixels have a lateral size of 1.038 *μ*m. On average, there are 240 cells per image. One expert created the gold standard by performing manual segmentation of the cell edges using an open-source image manipulation program (GIMP).

### U-net architecture

**Fig. 8:**
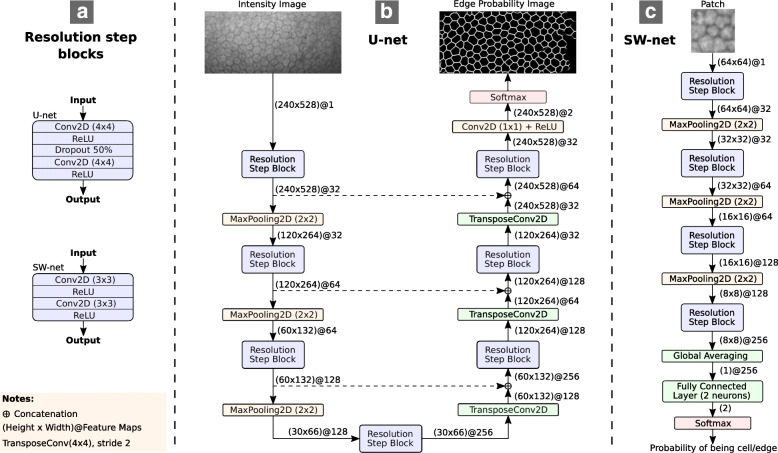
Schematic overview of U-net (**b**) and SW-net (**c**). The blocks summarizing the layers at the different resolution steps are indicated in (**a**). For U-net, the network consists of a contraction path and an expansion path. In contrast, SW-net is, in essence, the same contraction path of U-net with a global averaging layer and a fully connected layer of 2 neurons in the end

The U-net follows a standard fully convolutional architecture, with a contraction and an expansion path, each composed of four resolution steps (Fig. 8). In the contraction path, each step consists of two 4 ×4 padded convolutions with a rectified linear unit (ReLU), a dropout layer with a drop rate of 50% between the two convolutions, and a 2 ×2 max pooling with stride 2 at the end for downsampling. In the expansion path, each step contains a 4 ×4 transposed convolution with stride 2 for upsampling, a concatenation with the corresponding feature map from the contraction path, two 4 ×4 padded convolutions with ReLU, and a dropout layer with a drop rate of 50% between the convolutions. The convolutional layers in the first resolution step have 32 feature channels, doubling it at each downsampling step, and halving it at each upsampling step. In the last layer, a 1 ×1 convolution reduces the channels to the number of classes, which is set to two (cell body and cell edges). A cross-entropy loss function with a pixel-wise soft-max activation is used over the final feature map. No class weighting is employed. The optimizer of our choice is Adam [45] with an initial learning rate (*lr*_*i*=0_) of 0.001 and a decay of 0.001, such that *lr*_*i*_=*lr*_*i*−1_·(1/(1+decay·iteration)), where *i* denotes iteration. The network accepts the whole image as input. A batch size of 4 images is used.

Compared to the original U-net architecture, several modifications were introduced. First, we used a kernel size of 4 ×4 instead of 3 ×3, and the network was downscaled in width and depth, halving the number of feature channels and removing one resolution step.

Second, dropout layers were added in between the two consecutive convolutions per resolution step. Dropout is a regularization method used to avoid over-fitting, originally described for neural networks [46, 47]. It stochastically sets to zero a certain number of activations of hidden units at each training iteration. This prevents the co-adaptation of feature detectors by forcing neurons to rely on population behavior. In CNNs, it simply sets input values (of the feature maps) to zero.

Third, we used transposed convolutions for upsampling in the expansion path. The transposed convolution is described as the operation that forms the same connectivity as the normal convolution but in the opposite direction [48]. Since the weights in the transposed convolution are learnt, this avoids to predefine an interpolation method for upsampling. Unfortunately, transposed convolutions can also produce a checkerboard effect due to the uneven overlapping of the filter range in the output pixels [49]. Specifically, the uneven overlapping occurs when the kernel size is not divisible by the stride. While the CNN could, in principle, learn weights to avoid this problem, in practice this effect is often observed, especially in images with strong colors. One practical solution is to use a 4 ×4 kernel size with a stride of 2 [50]. Nonetheless, we did not observe in our work the checkerboard effect when using filters of 3 ×3 with a stride of 2.

### SW-net architecture

The SW-net architecture follows the same contraction path as the aforementioned U-net (Fig. 8). However, instead of the entire image, a patch of size 64 × 64 is provided as input, and the filter size of the convolutional layers is 3 ×3. At the end of the contraction path it adds a global averaging pooling layer, where each channel is reduced to its average value, and a fully connected layer of two neurons, which provides the outcome for the two classes regarding the central pixel of the patch. A batch size of 128 patches is used here, which holds a similar amount of data as the batch in our U-net. Moreover, the same loss function and optimizer is employed.

The original Cireşan et al.’s architecture [3] consisted of four stages of one convolutional layer followed by max-pooling. All convolutional layers had 48 feature maps and filters of size 4 ×4 (one of 5 ×5). The network ended with two fully connected layers: one of 200 neurons followed by another with 2 neurons to obtain the class labels. In comparison, our network has doubled the number of convolutional layers, albeit with a smaller kernel size, increasing the receptive field (61 pixels instead of 48 pixels). Moreover, we substituted the large fully connected layer with a global averaging pooling. This idea was originally suggested by Lin et al. [51], where he argued that fully connected layers at the end of a CNN are prone to over-fitting, whereas global averaging layers are more native to the convolution structure, over-fitting is avoided, and the feature maps can be interpreted as categories confidence maps.

### Prediction

For U-net, the segmentation was retrieved directly from the network output. In the SW-net, one patch per each pixel was retrieved, building up the segmentation image with the classification value of each patch. Images were mirrored in order to extract the patches that reached beyond the image borders.

### Postprocessing

**Fig. 9:**
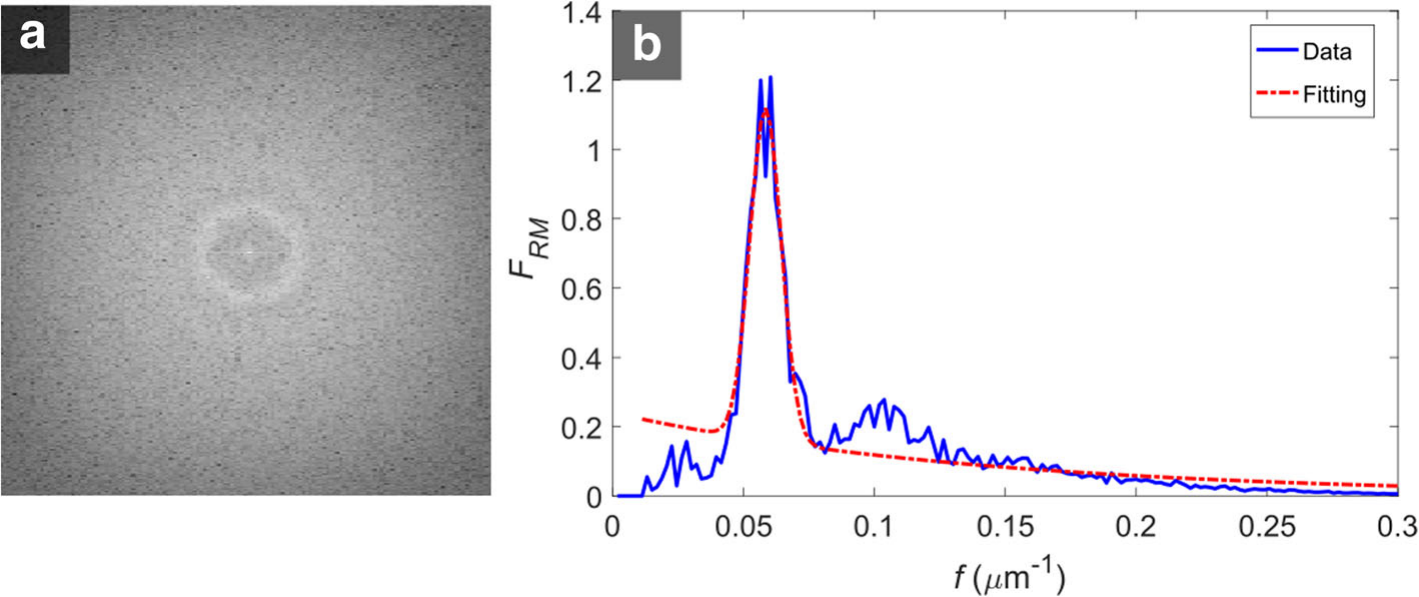
**a** 2D FT of the U-net output of a CE image (up to *f*=0.3). **b** The magnitude of the FT after reconstruction by dilation and angular averaging (blue), and the fitted model (red) in order to estimate the peak

To obtain the final segmentation, we smoothed the CNN output and applied the classic watershed algorithm [52]. Specifically, we first estimated the average cell size in the image by Fourier analysis in order to built a Gaussian smoothing filter whose standard deviation was related to that size. It is well known how the 2D Fourier Transform (FT) of a CE image shows a distinctive concentric ring due to the fairly regular pattern of the cells [27], and for the output of the CNN that ring is clearly noticeable (Fig. 9a). Selig et al. described in [27] how the radius of the ring, called *characteristic frequency* (*f*^∗^), is related to the most common cell size in the image, *l*=1/*f*^∗^. We estimated the radius by first applying a method called ‘reconstruction by dilation’ to remove the low frequencies (defined by Selig et al. in [27]) and later computing the 1D radial magnitude, defined as the angular averaging of the magnitude of the 2D FT of the images,

4 $$ \mathcal{F}_{RM}(f)=\frac{1}{2\pi}{\int\nolimits}_{0}^{2\pi}{|\mathcal{F}(f,\theta)|d\theta},   $$

where $\mathcal {F}(f,\theta)$ is the FT of the image in polar coordinates (Fig. 9b). In our previous work [39], we described a fitting function to estimate the peak position (*f*^∗^) and also derived a parameter, *k*_*σ*_=0.20, used to adapt the filter *σ* to each image, *σ*=*k*_*σ*_/*f*^∗^. Once images were smoothed, the watershed algorithm was applied, and the clinical parameters were estimated from the resulting images. The classic watershed does not require any parameter tuning, but it is expected that each object (cell) to detect has a single local minimum, otherwise cells will be oversegmented.

### Labels

**Fig. 10:**
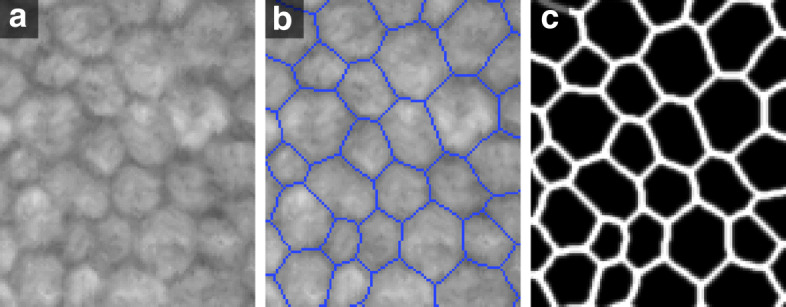
**a** Raw intensity image, size 120 ×100 pixels. **b** Gold standard superimposed on the image. **c** Label image

The gold standard, a binary image where value 1 indicates a cell edge and value 0 represents a cell body, was defined such that cell edges are 8-connected-pixel lines of 1 pixel-width (Fig. 10b). In the intensity image, the cell edges might appear thicker, with a steep but clear transition in intensity from the peak of the edge towards the inner cell. However, this thickness might vary considerably even in the same image (Fig. 10a). Hence, instead of using the gold standard images as labels, we proposed to use probabilistic labels where edges appear thicker and in which the aforementioned intensity transition between edges and cells is preserved. There are three reasons for doing so: (1) it is counterproductive to teach the network that the pixels adjacent to the annotated 1-pixel-width edge are cell pixels as they usually have the same characteristics as the annotated edge; (2) mimicking the intensity transition in the labels is a more natural approach and helps the network in its classification task; (3) as the network will learn to replicate this pattern (gradual intensity transition between edges and cell bodies), this will be beneficial when applying the watershed algorithm in the postprocessing step.

To create the probabilistic labels, we convolved the gold standard images with a 7 ×7 isotropic unnormalized Gaussian filter of standard deviation 1 pixel. This allowed all pixels with label 1 (edges) in the gold standard to keep a value equal to 1 in the probabilistic label image, with increasingly smaller probabilities for pixels further away from the annotated cell edge (Fig. 10c). Hence, the pixels in the label image can be regarded as the probability of being part of an edge. This is used as the target output of the networks to be trained. During evaluation, the edge class was considered any pixel with *p*>0.5. In practice this means that we accept a 1 pixel error in the location of the edge. For comparative purposes, we also evaluated the outcome segmentation when the ‘hard’, binary gold standard labels are used as target output.

### Preprocessing of the intensity images

**Fig. 11:**
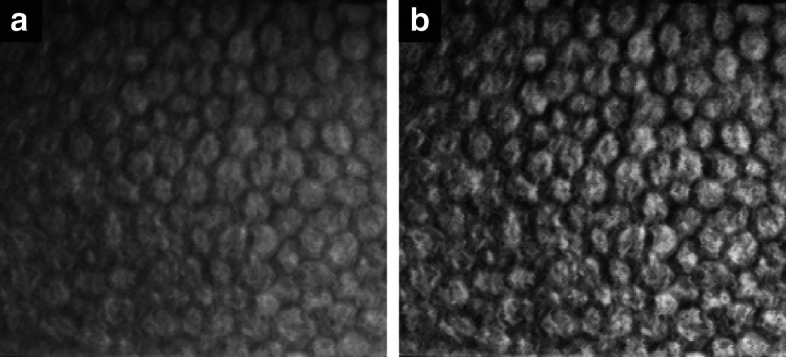
**a** Portion of a specular microscopy image, size 240 ×261 pixels. **b** The intensity image after CLAHE

Specular microscopy images usually have a non-uniform luminosity across the image and low contrast (Fig. 11a). Here, we want to evaluate whether the CNN can benefit from some kind of image enhancement. Furthermore, it is common practice in neural networks to standardize the input images,

5 $$ {image}_{stand} = \frac{\text{image} - mean(\text{image})}{std(\text{image})},  $$

or normalize them,

6 $$ {image}_{norm} = \frac{\text{image} - min(\text{image})}{max(\text{image})-min(\text{image})}.  $$

To enhance local image contrast, we proposed to use contrast limited adaptive histogram equalization (CLAHE) [36] with a kernel of 24 ×24 (Fig. 11b). This kernel size matches approximately the area of the average cell. A kernel with a size less than half of a cell would overamplify noise, whereas a kernel too large would reduce the benefits of local contrast enhancement. In earlier work on aneurysm detection in fundus images, we achieved a much better performance with intensity normalization than without it [53].

In summary, we tested the influence of preprocessing by analyzing five possible scenarios: feeding the raw images, normalizing them, standardizing them, and enhancing them by CLAHE (with and without standardization, since the output of CLAHE is already normalized).

### Data augmentation

Given the nature of the images, flipping them horizontally and/or vertically was a natural way of augmenting the training data by a factor of four. We avoided other transformations, such as rotation or elastic deformations [54], for two reasons: (1) the images show a small degree of distortions only in horizontal and vertical lines, hence rotating or deforming the images would create new noise patterns that do not exist in the original images; (2) when rotating, the image corners need to be filled, either by mirroring the image or setting that area in black; either way, we are introducing new patterns to be solved by the network.

### Implementation details, and computational cost

The data set was divided in 5 folds of 10 images each. To obtain the optimal network parameters, we used 4 folds for training and 1 for validation/test. For the evaluation of the CNN segmentation and the clinical parameters, a 5-fold cross-validation approach was employed in order to test all the remaining folds, using the parameters determined in the first test set.

Regarding class weighting in U-net, we evaluated whether adding weights in the loss function was advantageous. Here, the edge class has 4 times less pixels than the cell class. For the SW-net, we sampled the same amount of patches per class in each batch.

Other loss functions were tested, specifically mean-squared and mean-absolute loss, but with very similar performance as using cross-entropy. Batch normalization layers [55] were also tested by including them after every ReLU, but this created slightly more over-fitting and degraded the performance. Similarly to what Springenberg et al. reported in [56], no differences were observed in SW-net if max-pooling layers were substituted with a stride of 2 in the previous convolutional layer.

CNN filter weights were initialized from an uniform distribution of mean=0 and width≈1 (glorot uniform initializer in Keras). Networks were coded in Python 3.6 programming language, using the Keras library and Tensorflow as backend. Experiments were run in the free research tool Google Colaboratory, which includes GPU support (Tesla K80), taking roughly 0.8 s per training iteration in U-net and 0.5 s for the SW-net. The testing took less than 1 s per image for U-net. However, for the SW-net, evaluating all patches in an image took around 1 min. The postprocessing and parameter estimation took barely 1-2 s per image.

### Metrics and statistical analysis

In the evaluation of the CNNs performance, accuracy and AUC were provided. However, due to the probabilistic nature of the labels, pixels with label values *p* close to 0.5 are not relevant for our ultimate goal. Indeed, the most important pixels are either at the crest of the cell edge (*p*=1) or at the inner cell body (*p*=0). Furthermore, the class imbalance makes it important to evaluate each class performance independently. Hence, we also reported the precision (PRE), sensitivity (SEN), and specificity (SPE) for the final designs, but only considering the pixels with values 0 and 1 in the label images. For clarification purposes, we placed an asterisk (*) in the metrics that followed this rule.

In the evaluation of the postprocessed segmentation, only the cells within the area of the gold standard were kept, discarding all cells in contact with the image borders. We used the modified Hausdorff distance (MHD) [38] to measure the distance between the gold standard and the proposed segmentation. MHD is defined as

7 $$ \text{MHD}(\mathcal{U,V})=max(hd(\mathcal{U,V}), hd(\mathcal{V,U})),  $$

where

8 $$ hd(\mathcal{U,V})=\frac{1}{|\mathcal{U}|} \sum\limits_{a \in \mathcal{U}} \min_{b \in \mathcal{V}} ||a-b||_{2},  $$

$\mathcal {U}$ is the gold standard segmentation, and $\mathcal {V}$ the proposed segmentation.

DICE [31] was used to assess the segmentation at the cell level. We computed the DICE for each cell independently, reporting the average DICE. Specifically, for each cell (*C*_*i*_) in the gold standard images, we select the superpixel (*S*_*j*_) in the proposed segmentation with the largest overlap to *C*_*i*_, such that TP =*C*_*i*_∩*S*_*j*_ (True Positive), FN =*C*_*i*_∖*S*_*j*_ (False Negative), FP =*S*_*j*_∖*C*_*i*_ (False Positive),

9 $$ \text{DICE}_{ith \ cell}=\frac{2 \cdot TP}{2 \cdot TP + FP + FN},  $$

10 $$ \text{DICE}_{image}=\frac{1}{n} \sum\limits_{i=1}^{n} \text{DICE}_{ith \ cell},  $$

where *n* is the number of cells in the image.

We also evaluated the number of cells correctly segmented, reporting the number of cells that were oversegmented (divided in more than one superpixel) and undersegmented (within a superpixel that covers more than one cell). We considered a cell was correctly segmented if its *TP*_*i*_>0.80·*max*(*C*_*i*_,*S*_*j*_). That margin was added to allow small deviations in the cell boundary locations and was selected after visual analysis.

For the three previous metrics, either the parametric paired t-test or the non-parametric Wilcoxon signed-rank test was performed to determine which method, U-net or SW-net, was more accurate. We used the non-parametric test when the distributions did not fulfill the normality assumption (Shapiro–Wilk normality test). A *p*-value of *p*<0.05 was considered statistically significant.

In the evaluation of the clinical parameters, a statistical analysis based on linear mixed-effects models [57] was performed to determine, for each parameter, whether there was a statistically significant difference in accuracy (smaller absolute mean) and in precision (smaller variance) between the two estimation errors. To determine whether the variances were different, we used a likelihood test to compare a model that assumes equal variances between both estimation errors with a model that assumes different variances. From the fixed effects test of the models we evaluated whether the absolute mean values in both estimations were different. No correction for multiple testing was applied, and a *p*-value of *p*<0.05 was considered statistically significant.

## Abbreviations

AUC: Area under the ROC curve
CNN: Convolutional neural networks
CLAHE: Contrast limited adaptive histogram equalization
CE: Corneal endothelium
CV: Cell variation (polymegethism)
ECD: Endothelial cell density
FP: False positive
FT: Fourier transform
HEX: Hexagonality (pleomorphism)
MHD: Modified Hausdorff distance
PACG: Primary angle-closure glaucoma
POAG: Primary open-angle glaucoma
PRE: Precision
ReLU: Rectified linear unit
ROC: Receiver operating characteristic
SD: Standard deviation
SEN: Sensitivity
SPE: Specificity
SW-net: Sliding-window network
TP: True positive
TN: True negative
